# The use of medications approved for alcohol use disorders in Italy

**DOI:** 10.3389/fpubh.2023.1110435

**Published:** 2023-02-15

**Authors:** Filomena Fortinguerra, Andrea Pierantozzi, Francesco Trotta

**Affiliations:** Italian Medicines Agency (AIFA), Rome, Italy

**Keywords:** alcohol use disorders, drug use, disulfiram, sodium oxybate, acamprosate

## Abstract

**Background:**

Italy has the highest per capita alcohol consumption among European countries. Several pharmacological treatments for alcohol use disorders (AUDs) are currently available in Italy, but no consumption data are available. A first analysis of national drug consumption, comprising the whole Italian population over a long-term period covering the COVID-19 pandemic, was performed.

**Methods:**

To analyze the consumption of medications indicated for therapy of alcohol dependence, different national data sources were used. Consumption was measured as a defined daily dose (DDD) per 1,000,000 inhabitants per day.

**Results:**

In 2020, the total consumption of medicines used in the treatment of AUDs amounted to 310.3 DDD per 1 million inhabitants per day (0.018% of the overall drug consumption in Italy) with a decreasing gradient from the north (373.9 DDD) to the south (250.7 DDD). 53.2% of the overall doses were dispensed by public healthcare facilities and 23.5% by community pharmacies, while the remaining 23.3% were purchased privately. The temporal trend of consumption seemed to be stable across the last few years, although an impact of the COVID-19 pandemic was observed. Disulfiram was the most consumed medicine over years.

**Conclusion:**

All Italian regions offer pharmacological treatments to patients with AUDs, but differences in the number of dispensed doses suggest a different local organization of patient care, which can be partly explained by the different severity of the clinical condition of residing patients. Pharmacotherapy of alcoholism should be deeply investigated to describe the clinical characteristics of treated patients (i.e., comorbidities) and evaluate the appropriateness of prescribed medications.

## Introduction

Alcohol use disorders (AUDs) represent a major public health problem, affecting over 100 million individuals every year worldwide, with an overall prevalence of 1.3% in 2016 (1.7 among men; 1.0% among women), ranking among the most prevalent of all substance use disorders at the global level (1–3). The risk of AUDs and related mortality follows a socioeconomic gradient, with individuals of low socioeconomic status being at increased risk (4–7).

Per capita alcohol consumption in Europe is the highest in the world, being responsible for ~4% of all deaths and ~5% of the years of life lost due to ill health, disability, or early death (8).

In Italy, specific legislation is in place (9) which describes all the interventions to be implemented at the national level for the prevention, treatment, and rehabilitation of alcohol-related pathologies requiring an annual review on this topic by the Italian Parliament. The 2020 Italian Ministry of Health Report highlighted that 14.3% of the Italian population over 10 years old were at increased risk of developing alcohol-related health harms. Furthermore, the prevalence of adults (>17 years) with a harmful consumption of alcohol to be considered “in need of treatment” (10) and clinically assimilated to alcohol dependence was 2.3% in men and 1.1 in women (11).

Pharmacological treatments for alcohol dependence are available aiming at the maintenance of complete abstinence and the recovery of psychophysical and social skills and cover different phases from acute alcoholic intoxication to alcohol withdrawal syndrome (12). However, in Europe, the treatment rates for AUD are generally low (<20%), partly because of the high stigma associated with the disease (13–16).

According to the international guidelines relating to the pharmacological treatment of patients with alcohol dependence (17–22), pharmacotherapy should be considered on a routine basis for all patients with moderate-to-severe alcohol use disorders in addition to evidence-based psychosocial therapies, professional counseling, and mutual-help group support. The main goals of pharmacological treatment of alcohol dependence or the prevention of relapse to alcohol use are either abstinence or reduction of heavy drinking. In general, the duration of the treatment program should be at least 6–12 months. However, since AUD is a chronic medical problem, patients may need to use medications for a longer period or may require multiple episodes of pharmacotherapy.

Based on available clinical guidelines, acamprosate or naltrexone may be offered as first-line treatments to patients with moderate-to-severe AUD. These medications have a well-established efficacy and can be also used in combination with psychotropic medications. Due to difficulties with adherence and toxicity, disulfiram is recommended as a second-line option and should be reserved for patients who have no contraindications to use it (liver cirrhosis or psychosis), with high motivation to maintain abstinence, or are intolerant to or non-responder to naltrexone or acamprosate.

Sodium oxybate, approved for AUD treatment only in few European countries, such as Italy, Austria, and France, acts as an alcohol substitute as a kind of replacement therapy, reducing craving for alcohol and preventing alcohol withdrawal syndrome. In Italy, it is used in the hospital management of the alcohol withdrawal syndrome for a short term only (7–10 days), while the prolonged treatment of AUDs for the prevention of relapses and the maintenance of abstinence is not approved. This medication should be prescribed to severe alcohol-dependent patients with a very high drinking risk level associated with psychiatric comorbidities (22–25). However, it should be noted that other authors reported different conclusions about the use of sodium oxybate in patients with psychiatric comorbidities (23, 26). In particular, in some subtypes of alcoholic patients, e.g., those affected by co-addiction to heroin or cocaine and/or psychiatric comorbidity, such as borderline personality disorders, sodium oxybate appears to be at high risk of developing a craving for and abuse; therefore, it may not be indicated in these patients.

For the treatment of alcohol withdrawal syndrome and/or alcohol-related symptoms (hallucinations, agitation, convulsions, depression, restlessness, and insomnia), other classes of drugs are (co)-administered, such as benzodiazepines, antipsychotics, antidepressants, and antiepileptics (27).

Patients can be treated by a general practitioner if they agree to abstain from alcohol, or in case of serious medical, social, or psychiatric complications as well as in case of treatment failure can be referred to specialists (27, 28).

Despite AUDs being a relevant public health issue in our country, to date, neither national nor regional studies on drug consumption and expenditure for AUDs treatment have been published in Italy. A few other European countries, such as Norway (29), Sweden (30, 31), Germany (32), and the UK (33), published studies describing the use of drugs for alcohol dependence, but they were not updated or involved a small sample of patients or those with specific characteristics (such as patients admitted to hospital for AUD).

The aim of this article was to describe the use of drugs for alcohol dependence use in Italy, based on national consumption data, also providing details on the type of medicine, type of assistance, geographical distribution, and expenditure. Temporal trends were analyzed to better evaluate the impact of the COVID-19 pandemic on the use of this class of medicines.

## Methods

The consumption and expenditure data for medicines authorized in Italy for AUDs and dispensed both by community pharmacies and public health facilities were collected during the period 2016–2020. Data cover the medicines dispensed to the whole Italian resident population either reimbursed by the Italian National Health Service (NHS) or privately purchased.

Medicines included in the analyses were disulfiram (ATC N07BB01), sodium oxybate (ATC N07XX04), acamprosate (ATC N07BB03), naltrexone (ATC N07BB04), nalmefene (ATC N07BB05), and metadoxine (ATC N07BB).

Drug consumption was defined as the number of defined daily doses (DDDs), which is the assumed average maintenance dose per day for a drug used for its main indication in adults.

The consumption and expenditure analyses are also expressed based on the resident population, respectively, in “DDD per 1,000,000 inhabitants per day” and “euros per 100 inhabitants,” to allow regional comparisons. The compounded average growth rate (CAGR) was calculated to estimate the evolution of drug use over time. In addition, the average cost per DDD was also calculated.

Analyses were conducted by stratifying consumption and expenditure data by geographical area, active ingredient, and year. For the impact analysis of the COVID-19 pandemic on AUD drug treatments, monthly consumption data during 2019–2021 were used.

## Results

In Italy, there are six drugs authorized for the treatment of AUDs; five medications (disulfiram, acamprosate, naltrexone, metadoxine, and nalmefene) can be prescribed by a general practitioner, and one (sodium oxybate) should be used under medical supervision in a specialist setting only.

During 2020, the total consumption of medicines used in the treatment of AUD amounted to 310.3 DDD per 1 million inhabitants per day (0.018% of the overall drug consumption in Italy), with a total expenditure of 11.3 euros per 100 inhabitants and a total expenditure of 6.75 million euros (0.022% of the overall drug expenditure in Italy) (34) (Table 1). A sum of 86.8% of the total expenditure is public (through the Italian NHS) corresponding to 76.7% of the consumption, while the remaining part represents private spending (23.2%).

**Table 1:** Consumption and expenditure for medicines with the main therapeutic indication relating to the treatment of alcoholic disorders in Italy during 2020.

	**Community pharmacies**			**Public healthcare facilities**			**Reimbursed by the Italian NHS (community pharmacies** + **public healthcare facilities)**			**Private purchase**			**Overall**	
	***N***.	**%**	Δ**% 20–19**	***N***.	**%**	Δ**% 20–19**	***N***.	**%**	Δ**% 20–19**	***N***.	**%**	Δ**% 20–19**	***N***.	**%**
**Consumption (DDD/1.000.000 inhabitants per day)**														
North	75.3	20.2	−16.5	216.9	58.0	−14.2	292.2	78.2	−15.4	81.7	21.8	−21.7	373.9	100
Center	96.9	37.5	−7.1	104.0	40.3	−20.5	200.9	77.8	−13.8	57.4	22.2	−19.1	258.3	100
South	54.6	21.8	−9.8	128.4	51.2	−28.8	183.0	73.0	−19.3	67.7	27.0	5.8	250.7	100
**Italy**	**72.9**	**23.5**	–**12.5**	**165.3**	**53.2**	–**19.3**	**238.2**	**76.7**	–**17.5**	**72.2**	**23.3**	–**14.4**	**310.3**	**100**
**Expenditure (**€ **per 100 inhabitants)**														
North	1.4	13.1	−15.1	7.7	72.2	−29.4	9.1	85.3	−22.3	1.6	14.7	−25.1	**10.6**	100
Center	3.9	34.7	1.5	6.0	53.6	−26.5	9.9	88.3	−12.5	1.3	11.8	−44.9	**11.2**	100
South	1.0	7.8	−3.2	10.0	80.2	−32.8	11.0	88.0	−18.0	1.5	12.0	−5.3	**12.5**	100
**Italy**	**1.7**	**15.5**	–**5.6**	**8.1**	**71.3**	–**30.4**	**9.8**	**86.8**	–**18.0**	**1.5**	**13.2**	–**24.7**	**11.3**	**100**

In total, 53.2% of the doses were dispensed by public healthcare facilities, while 23.5% were dispensed by community pharmacies; the remaining 23.3% of the doses were purchased privately by the patients.

Considering the overall consumption by geographical areas, a decreasing gradient was observed from the northern area (373.9 DDDs/1,000,000 inhabitants per day) to the south (250.7 DDDs). In all areas, the majority of doses were dispensed by public healthcare facilities (north 58.0%, center 40.3%, and south 51.2%). The central area had the highest percentage of doses dispensed in the community pharmacies (37.5%), while the south had the highest percentage of doses purchased privately by patients (27.0%). The northern area where the highest consumption was observed had the highest coverage by the Italian NHS (78.2%) and the lowest percentage of doses purchased privately (21.8%).

The overall Italian NHS expenditure for medicines for the treatment of AUDs in Italy was similar throughout the three geographical areas (ranging between 85.3 and 88.3 euros per 100 inhabitants). The southern area had a higher expenditure (10 euros per 100 inhabitants) for medicines dispensed by public healthcare facilities than other areas, although the number of doses delivered is half of those in the north (128.4 vs. 216.9 DDDs). Furthermore, for medicines dispensed by community pharmacies, the central area had an expenditure almost three times greater than in the northern regions (3.9 euros vs. 1.4 euros per 100 inhabitants) and four times greater than those of the south (1.0 euro), although it should be considered that dispensed doses by the community pharmacies in the central area were almost double when compared to the south and a half more compared to the north. Regarding private spending, no difference was found between the north, central, and south areas (ranging between 1.3 and 1.6 euros per 100 inhabitants).

During 2020, a strong decrease was observed in the overall drug consumption and expenditure at the national level compared to 2019, respectively, −17.5 and −18.0% for drugs reimbursed by the Italian NHS; similarly, a strong decrease in the private purchase was observed during 2020 (−14.4% in doses and −24.7% in spending).

The reduction in reimbursed doses and public expenditure was less pronounced in the central area compared to the north and the south; the private purchases can be considered stable in the south when compared with the strongest reduction in the central and the north.

Considering the 5-year period 2016–2020, a negative trend in the consumption of medicines reimbursed by the Italian NHS was observed (CAGR −4.3%) (Table 2). However, in 2020, the highest decrease in overall consumption was observed when compared to 2019 (−17.5%). This decrease was stronger for drugs dispensed by public healthcare facilities (−20.1%) than those dispensed by community pharmacies (−10.9%). Considering the period 2016–2019, a smaller variation in overall drug consumption reimbursed by the Italian NHS was observed, highlighting a stable overall consumption over the pre-COVID-19 years.

**Table 2:** Annual pharmaceutical consumption (N. DDD/1,000,000 inhabitants per day) for treatment of alcoholic disorders during the 2016–2020 period.

**Year**	**Community pharmacies**			**Public healthcare facilities**			**Reimbursed by the Italian NHS (community pharmacies** + **public healthcare facilities)**		
	***N***.	**%**	Δ**% previous year**	***N***.	**%**	Δ**% previous year**	***N***.	**%**	Δ**% previous year**
CAGR 16–20	−3.6%			−5.2%			−4.3%		
2016	84.7	29.2	–	205.0	70.8	–	289.7	100	–
2017	97.2	31.7	14.7	209.3	68.3	2.1	306.5	100	5.8
2018	90.1	31.1	−7.3	199.5	68.9	−4.7	289.6	100	−5.5
2019	81.8	28.3	−9.2	206.8	71.7	3.7	288.6	100	−0.4
2020	72.9	30.6	−10.9	165.3	69.4	−20.1	238.2	100	−17.5

CAGR, compound annual growth rate.

During 2020, disulfiram covered 60.4% of the overall dispensed doses for the AUDs' medicines, accounting for 53.1% of doses reimbursed by the Italian NHS; sodium oxybate covering 21.1% of the overall dispensed doses was the second most dispensed medicine, used exclusively within public healthcare facilities (accounting for 27.5% of the publicly reimbursed AUDs' medicines); acamprosate was the third most consumed medicine covering 9.8% of the overall dispensed doses, mostly reimbursed by the Italian NHS. The first three drugs account for 91.3% of the overall dispensed doses in Italy (both public and private purchases) (Table 3).

**Table 3:** Pharmaceutical consumption (N. DDD/1,000,000 inhabitants per day) by medicine during 2020.

	**Community pharmacies**			**Public healthcare facilities**			**Reimbursed by the Italian NHS (community pharmacies** + **public healthcare facilities)**			**Private purchase**			**Overall**		
	***N***.	**%**	Δ**% 20–19**	***N***.	**%**	Δ**% 20–19**	***N***.	**%**	Δ**% 20–19**	***N***.	**%**	Δ**% 20–19**	***N***.	**%**	Δ**% 20–19**
Disulfiram	52.4	71.9	−14.9	74.0	44.8	−3.5	126.3	53.1	−8.6	61.2	84.8	−11.3	187.5	60.4	−9.6
Sodium oxybate	–	–	–	65.5	39.6	−36.6	65.5	27.5	−36.6	–	–	–	65.5	21.1	−36.8
Acamprosate	12.1	16.6	−8.1	17.0	10.3	−0.7	29.1	12.2	−0.9	1.4	1.9	−70.7	30.5	9.8	−11.6
Naltrexone	8.4	11.5	3.5	6.1	3.7	9.4	14.4	6.1	7.1	2.7	3.7	−7.9	17.2	5.5	4.2
Metadoxine	< 0.05	~0	~0	2.5	1.5	−10.2	2.6	1.0	−10.3	6.2	8.6	−8.4	8.7	2.8	−8.4
Nalmefene	< 0.05	~0	~0	0.2	0.1	−80.6	0.3	0.1	−80.1	0.7	1.0	−24.5	0.9	0.3	−55.0
Total	72.9	100	−10.9	165.3	100	−20.1	238.2	100	−17.5	72.2	100	−14.4	310.4	100	−16.9

An overall decrease of 16.9% in consumption was observed during 2020 for the whole category of AUDs' medicines, mostly due to the decrease in the number of sodium oxybate doses (−36.8%), dispensed exclusively by public healthcare facilities; naltrexone was the only medicine which increased in consumption (+4.2%) during 2020. The consumption of both metadoxine and nalmefene dispensed exclusively by public healthcare facilities was negligible, accounting for 2.5 and 0.1%, respectively. The most part of the overall private spending was for disulfiram (84.8%).

**Figure 1:**
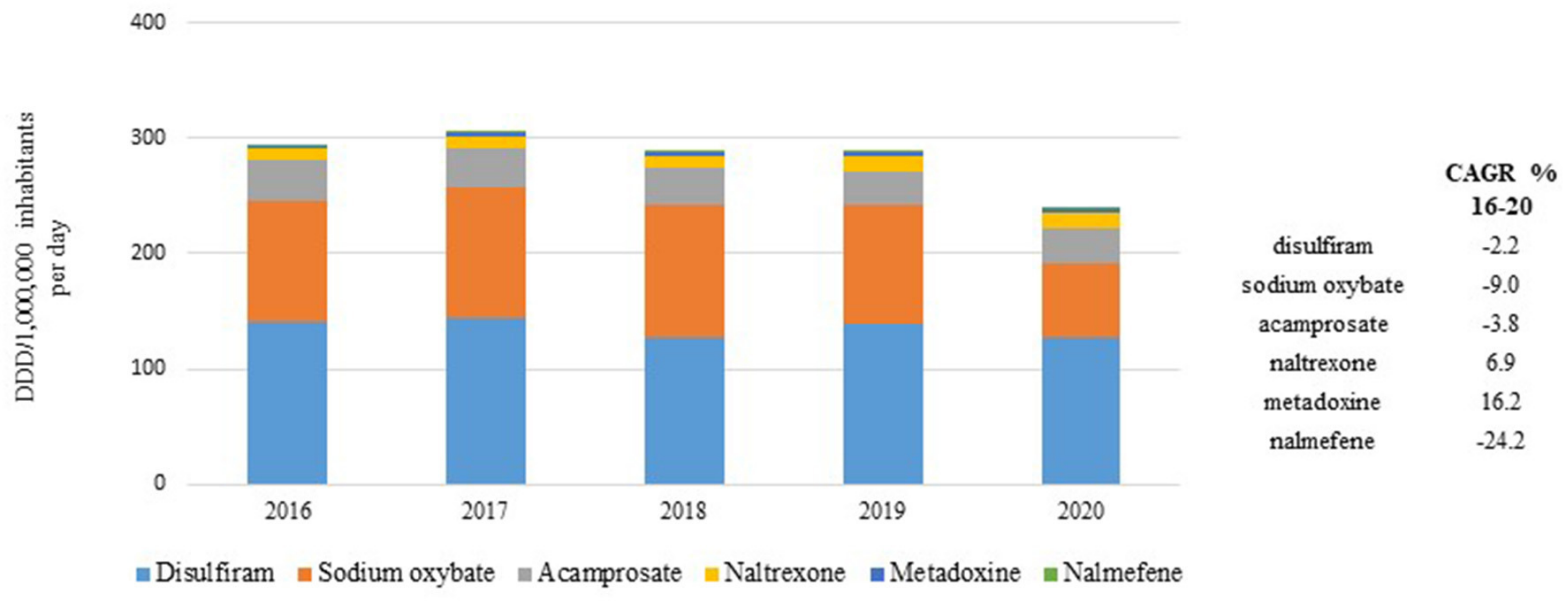
Temporal trend of consumption (N. DDD/1,000,000 inhabitants per day) for medicines reimbursed by the Italian NHS during 2016–2020 period.

Despite the strong decrease observed in 2020, the temporal trend of the overall drug consumption was stable over years up to 2019 with almost 300 DDD/million inhabitants per day; moreover, the proportion of the first three drugs most used (disulfiram, oxybate, and acamprosate) was also stable over the 4-year pre-pandemic period 2016–2019 (Figure 1). Considering the 5-year period 2016–2020, a negative temporal trend was observed in the consumption of sodium oxybate (CAGR −9.0%), acamprosate (CAGR −3.8%), and disulfiram (CAGR −2.2%), while the consumption of naltrexone increased over the years (CAGR +6.9%).

The average cost per daily dose of the whole category was 0.97 euros per 100 inhabitants; sodium oxybate (1/4 of consumption) has the highest cost per day (2.6 euros) followed by acamprosate with half of consumption (1.8 euros), while the cost of disulfiram, a generic drug, was very low (0.2 euros) (Table 4). Overall, despite a strong geographical variability in the number of dispensed doses, a low variability was found in the average cost per daily dose (ranging from 0.91 to 1.01 euros per 100 inhabitants); acamprosate (ranging from 1.6 to 2.1 euros) and naltrexone (ranging from 1.0 to 1.7 euros) registered the highest differences, while disulfiram and sodium oxybate, the most dispensed medicines, have no difference. The cost of nalmefene was the highest (4.1 euros) and most variable between geographical areas (ranging from 4.1 to 5.1 euros) with negligible consumption (0.1%).

**Table 4:** Consumption (N. DDD/1,000,000 inhabitants per day) and average cost per DDD (€) for medicines reimbursed by the Italian NHS during 2020 by geographical area.

	**Disulfiram**			**Sodium Oxybate**			**Acamprosate**			**Naltrexone**			**Metadoxine**			**Nalmefene**			**Overall**		
	***N***.	**%**	€	***N***.	**%**	€	***N***.	**%**	€	***N***.	**%**	€	***N***.	**%**	€	***N***.	**%**	€	***N***.	**%**	€
North	193.3	66.1	0.2	54.1	18.5	2.7	28.2	9.7	1.8	14.1	4.8	1.0	2.4	0.8	0.7	0.09	< 0.05	4.1	**292.2**	**100**	**0.99**
Center	87.8	43.7	0.2	57.1	28.4	2.6	29.2	14.5	2.1	26.3	13.1	1.7	0.6	0.3	0.8	< 0.05	< 0.05	5.1	**200.9**	**100**	**1.01**
South	53.8	29.4	0.2	87.3	47.7	2.6	30.2	16.5	1.6	7.6	4.2	1.3	3.7	2.0	1.1	0.5	0.3	4.1	**183.1**	**100**	**0.91**
Italy	**126.3**	**53.1**	**0.2**	**65.5**	**27.5**	**2.6**	**29.1**	**12.2**	**1.8**	**14.4**	**6.1**	**1.3**	**2.5**	**1.0**	**0.9**	**0.2**	**0.1**	**4.1**	**238.2**	**100**	**0.97**

**Figure 2:**
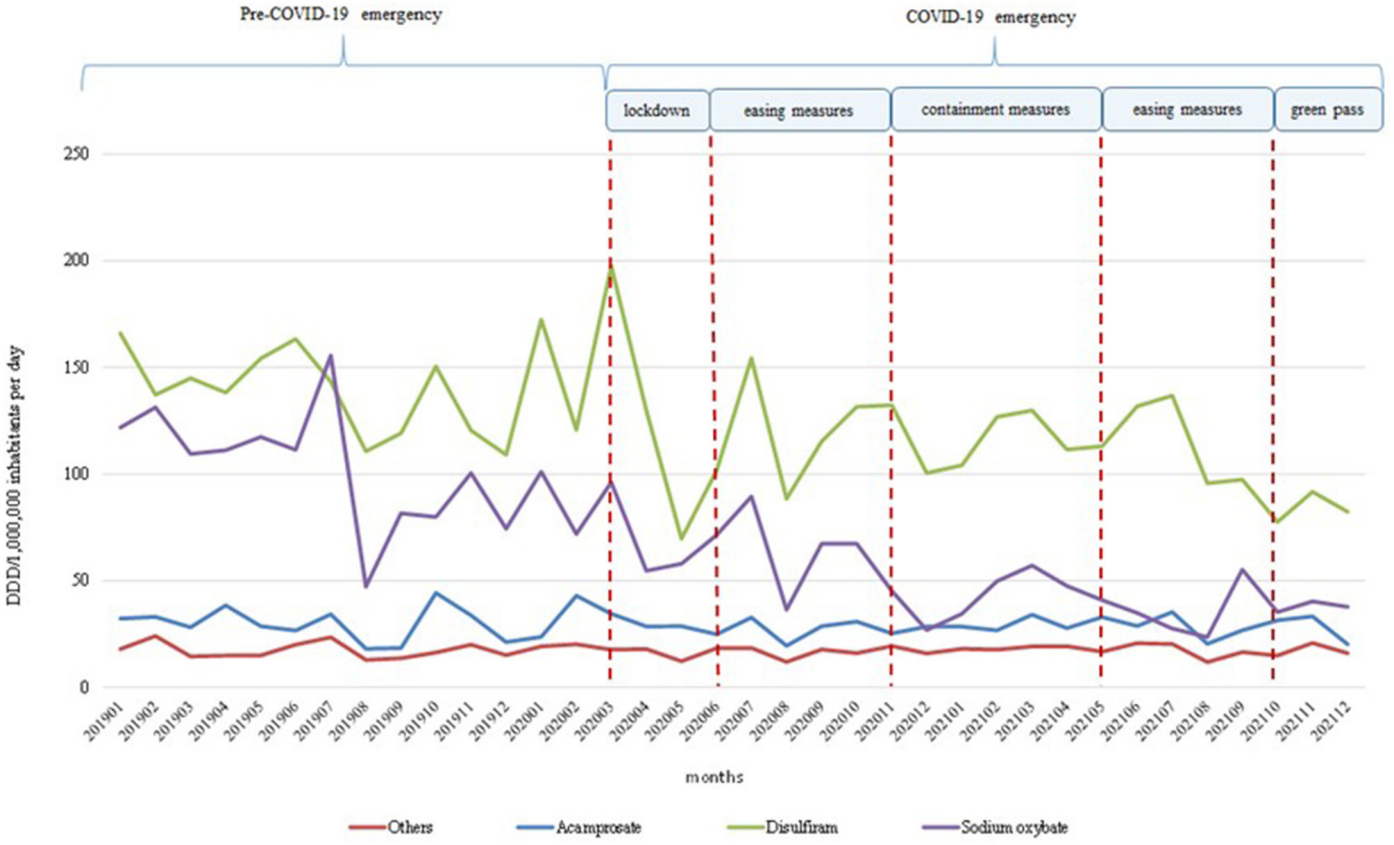
Temporal trends of consumption (N. DDD/1,000,000 inhabitants per day) of medicines reimbursed by the Italian NHS in 2019–2021 period.

An in-depth analysis of the monthly trends of drug consumption reimbursed by the Italian NHS during the 2019–2021 period (Figure 2), covering the COVID-19 pandemic, highlighted overall stability in dispensed doses during the pre-COVID-19 pandemic (January 2019–February 2020) for all AUDs' medicines but sodium oxybate which start decreasing from September 2019. A strongly decreasing during the lockdown period (March 2020–May 2020) was observed for disulfiram and sodium oxybate; then, a slight increase was observed for both, but they did not reach the pre-COVID-19 levels despite the easing and containment measures. Consumptions for acamprosate and others AUDs' medicines seemed weakly influenced by the COVID-19 pandemic.

## Discussion

This is the first study conducted in Italy describing the total consumption and expenditure for the six medicines approved for the treatment of alcohol dependence. We found a very low medicine consumption in 2020, ~310 doses per 1 million inhabitants per day considering both public and private purchases. At the national level, over 75% of the doses were reimbursed by the Italian NHS, with a proportion of 1:2 between community pharmacies and public healthcare facilities, while almost a quarter of the remaining dispensed doses are purchased privately by patients. Wide differences in the overall drug consumption were found between geographical areas; the north consumed more doses than the central and south areas, the central area dispensed more doses through the community pharmacies, and the south purchased more doses privately. The strong geographical variability in the number of dispensed doses between the various medicines may suggest a different organization of care for patients with AUDs between geographical areas, which can only be partly explained by the different severity of the clinical condition of patients residing in each area. Despite this, a low variability was found in the average cost per daily dose and expenditure both overall and for each medicine, thus suggesting a comparable purchasing capacity for these medicines throughout the national territory.

Disulfiram, an old generic drug, was the most consumed medicine in Italy over years. Sodium oxybate and acamprosate, medicines still under patent and costing more than disulfiram, had an overall consumption lower than disulfiram (21.1 and 9.8%, respectively). According to our results, a newer medicine, sodium oxybate, was often prescribed for the treatment of alcohol dependence. This framework suggested that the pharmacotherapy of alcoholism in Italy should be deeply investigated to describe the clinical characteristics of treated patients (i.e., comorbidities) and to evaluate whether the prescribed medications were appropriate according to the available clinical guidelines. For example, the high consumption of disulfiram as well as the adherence to naloxone or nalmefene treatments should be further investigated.

The highest decrease in overall consumption was observed in 2020 when compared to 2019 (−17.5%) probably because of the COVID-19 outbreak at the beginning of 2020; this hypothesis seems confirmed by the monthly temporal trend of consumption by medicine during 2019–2021, highlighting a stronger decrease for disulfiram and sodium oxybate. Probably, the measures of containment of the COVID-19 pandemic adopted in Italy affected the drug supply for sodium oxybate and disulfiram but not that for other drugs. This is because these medicines were mostly dispensed by public healthcare facilities, which were strongly engaged in dealing with the pandemic emergency, even in the period following the lockdown.

This study provided extensive data on the overall consumption and expenditure of all AUD medicines used in Italy, both supplied by NHS and purchased privately by patients, showing an exhaustive framework of the pharmaceutical assistance deliverable to the resident population on the entire national territory.

In this context, this is not a problem of cost or expenditure but a treatment gap and/or appropriate treatment prescribed to patients affected by alcohol dependence. Despite the efficacy, safety, and cost-effectiveness of the available medications, clinicians are reluctant to prescribe them to treat individuals with AUD. Insufficient knowledge, lack of access to care, and the few resources available in the management of the problem are major causes of delays in the diagnosis, finally contributing to significant clinical consequences that are much harder and costly to handle (35).

Currently, not all the Italian regions are compliant with the specific legislation in place (Law n. 125/2001), making it difficult to achieve the objectives set by the National Plan on Alcohol and Health. Although the plan has been active since 2007, the recognition of the current local strategies to counter alcohol addiction is still incomplete. Exploring some aspects of the treatment that have not been addressed is crucial to better evaluate the treatment offered both in primary care settings and specialized services for alcohol dependence. This unpleasant situation in Italy is the direct consequence of the failure to implement what was suggested (but not still implemented) by the law on alcohol and alcohol-related problems about the introduction in the university system of teaching on how to deal with substances and behavioral addictions; therefore, physicians answer AUD questions in a non-homogeneous way or delegate to other services or to the private-social sphere that is not recognized for its competence (21).

In general, our analysis confirmed the undertreatment of AUDs in Italy with an increasing gradient by geographical area. These data were in line with what is known about drug therapy to promote abstinence in alcohol-dependent patients, with the majority of patients receiving non-pharmacological therapy. However, studies from other European countries differ from our study because they are based on population-based registries (30, 31, 36) or healthcare databases covering small proportions of the national population (32, 34), while we focused on consumption and expenditure related to the overall national population. Only Norwegian data obtained from the National Prescription-Based Medicines Register (29) evaluated the complete information at the individual level about all prescription drugs dispensed from community pharmacies in Norway, even if drug treatment dispensed in hospitals or public healthcare specialist facilities was not included in the study, as the prescription register does not have such data at the individual level.

To our knowledge, this is the first Italian analysis on the consumption of medicines for the treatment of alcohol dependence in Italy, over a long-term period that also covers the COVID-19 pandemic. The strength of our study is the availability of medication consumption data covering the whole Italian population. However, no individual data were available; consequently, we were not able to fully investigate the drug prescription data by sex and age group, to identify the population subgroups most prescribed, the comorbidities and concomitant therapies, and the duration of treatment.

## Conclusion

Several medicines indicated for the pharmacotherapy of AUDs are available in Italy. They are reimbursed by the Italian NHS and dispensed nationwide by community pharmacies and public healthcare facilities. In all Italian regions, there is the possibility to offer pharmacological treatment to patients with AUDs, but the implementation of comprehensive and effective pharmacotherapy for all patients remains still a priority. Drug consumption was very low nationwide, and wide differences between geographical areas were found. The temporal trend of medication consumption seemed to be stable across the last 6 years, although an impact of the COVID-19 pandemic was observed.

Monitoring drug prescriptions for AUDs and investigating the social and clinical characteristics of treated patients (such as lifestyle and comorbidities), the evaluation of both appropriate use of medications and outcome associated with the pharmacological interventions, are urgently needed, especially in light of the available evidence showing an increased alcohol consumption during the COVID-19 pandemic (37–40). Given the high rate of relapse with psychosocial intervention alone, increasing patient access to underutilized treatments has the potential to improve clinical outcomes in this difficult-to-treat population. Furthermore, pharmacological treatment should be continued for a long-term period and should offer patients personalized care integrated for common comorbidities such as mental disorders. Guaranteeing the homogeneity of treatments and a qualitative improvement in the pharmaceutical assistance of patients affected by alcohol dependence, it will be associated with considerable health benefits across all geographical areas to reduce the significant psychosocial and public health consequences related to this important health problem.

## Data availability statement

Data were analyzed under license and are not available for public sharing.

## Ethics statement

Ethical review and approval and written informed consent for participation was not required for this study on human participants in accordance with the national legislation and institutional requirements.

## Author contributions

FT and FF contributed to the study conception. AP performed data collection and data analyses. FF drafted the manuscript. All authors contributed to interpretation of data, read, commented, revised, and approved the final version of the manuscript.
